# Personalized medicine at a prime time for cancer medicine – Introducing *Cancer Medicine*

**DOI:** 10.1002/cam4.1

**Published:** 2012-04-04

**Authors:** Qingyi Wei


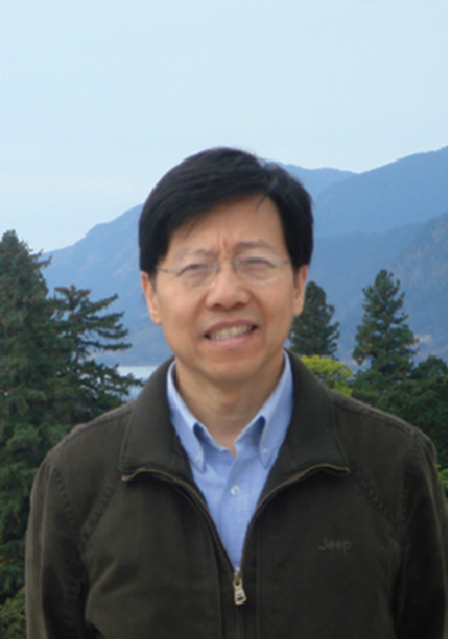


We are entering an important era when cancer medicine is being transformed into personalized medicine. To date, we have seen great advances in our understanding of the etiology, diagnosis, treatment and prevention of cancer, yet there have not been consistent declines in cancer incidence or death rates. One of the major hurdles in identifying the “magic bullet” for cancer, a term first used by Paul Ehrlich to describe the chemotherapeutic agent Salvarsan, is that each patient's cancer is complex and unique, with a distinct underlying molecular mechanism and targets for magic bullets. A personalised approach to cancer medicine is emerging with the goal of uncovering the unique pathway in each patient to tailor their therapy, thereby improving its success rate. Therefore, personalized cancer medicine represents a critical milestone and an integral part of modern cancer medicine.

Regarding etiological research, much progress has been made in genome-wide association studies in addition to pathway-based and candidate gene-based research, leading to the development of risk prediction models. It is inevitable that whole-genome sequencing will extend the current limits to personalized genetic cancer risk profiling. All these developments and advances are the key to the success of cancer medicine. For cancer diagnosis, tumor features at a genome-wide level can be depicted genetically and epigenetically, which can be further enhanced in animal models. These features open the door not only to identifying a cancer genetic signature for diagnosis but also to developing many alternative therapies for cancer that could enhance treatment efficacy, targeted therapies in particular. All of these achievements in research of etiology, diagnosis, and treatment will lead to effective identification of populations at risk, allowing for primary prevention as well as earlier diagnosis for secondary prevention.

At this time, I am delighted to introduce *Cancer Medicine*, a new Wiley Open Access interdisciplinary journal, which is committed to rapidly disseminating cutting-edge research and will consider submissions from all oncologic specialties, including, but not limited to, the areas of cancer biology, clinical cancer research, and cancer prevention to advance the personalized care of cancer patients.

*Cancer Medicine* will also cover aspects of science and technology in modern medicine, including basic research in cancer biology that uncovers tumor characteristics, clinical cancer research that promises the best therapies for the time being, and cancer prevention that aims at reducing cancer risk and occurrence. In this way, *Cancer Medicine* will allow the readers to have a fast access to the most updated global collaborations in cancer research and international approaches in practicing cancer medicine as well as achievements from the integration of basic, clinical, and preventive research of cancer.

*Cancer Medicine* will publish original research articles, systematic reviews, meta-analyses, and research methods papers, along with invited editorials and commentaries. Original research papers must report rigorous research with conclusions supported by the data presented in the paper. We are mindful of the need to maintain a high standard of data integrity and all manuscripts submitted to *Cancer Medicine* will be subject to thorough double-blind peer review by at least two referees. *Cancer Medicine* also works in partnership with other journals published by Wiley-Blackwell, including those owned by the world's most prestigious cancer societies, to give authors of high-quality work which cannot be accommodated in these leading journals the opportunity to transfer their manuscript for consideration by *Cancer Medicine*.

I am privileged to be supported by an outstanding editorial board of eminent researchers and clinicians who bring extensive and diverse expertise to the journal. In the years to come, I and the editorial board will work closely with the Wiley-Blackwell *Cancer Medicine* team to provide a timely and impartial service to all contributors and readers, who will, without a doubt, make *Cancer Medicine* a success.

